# Reciprocal relationships between sleep quality, mental health and the quality of life in elite athletes: A pilot study

**DOI:** 10.5114/biolsport.2026.154147

**Published:** 2025-09-09

**Authors:** Mohamed Romdhani, Emna Bentouati, Rihab Abid, Imen Moussa-Chamari, Karim Chamari, Helmi Ben Saad, Tarak Driss, Nizar Souissi

**Affiliations:** 1 Interdisciplinary Laboratory in Neurosciences, Physiology and Psychology: Physical Activity, Health and Learning (LINP2), UFR STAPS (Faculty of Sport Sciences), Paris Nanterre University, Nanterre, France; 2 High Institute of Sport and Physical Education, University of Sfax, Sfax, Tunisia; 3 Physical activity, Sport and health, UR18JS01, National Observatory of Sports, Tunis, Tunisia; 4 High Institute of Sport and Physical Education, ISSEP Ksar-Said, Manouba University, Tunis, Tunisia; 5 Laboratoire ACTES, UFR-STAPS, Université des Antilles, Pointe à Pitre, France; 6 Sport Coaching Department, College of Sport Sciences, Qatar University, Doha, Qatar; 7 Research Department, Naufar, Wellness and Recovery Center, Doha, Qatar; 8 Laboratoire de Recherche (LR12SP09) “Insuffisance Cardiaque” Sousse, Faculté de Médecine de Sousse, Hôpital Farhat Hached, Université de Sousse, Sousse, Tunisie

**Keywords:** Health Survey, Highly Trained Athletes, Mental Health, Recovery, Sleep Behavior

## Abstract

We aim to investigate the relationship between sleep quality, psychological health, and quality of life (QOL) in highly trained athletes. Elite athletes (n = 118, 20.1 ± 0.64 years; 39 females; 50 world class; 102 aged ≤ 25 years; and 76 practicing individual sports) responded to the Pittsburgh sleep quality index (PSQI), insomnia severity index (ISI), Epworth sleepiness scale (ESS), Depression, anxiety and stress scale (DASS), world health organization QOL (WHOQOL), and bespoke questions related to sleep hygiene. High percentages of the sample reported low or very low sleep quality (62%), moderate or excessive daytime sleepiness (51%), sleeping 7 hours or less (60%), and moderate or severe insomnia (16%), implying a modest sleep health. Female athletes reported higher PSQI (p < 0.05; d = 0.25), ESS (p < 0.05; d = 0.37) and DASS (p < 0.05; d = 0.27) scores compared to males. Young athletes (i.e., ≤ 25 years) reported higher PSQI (p < 0.05; d = 0.49) and DASS (p < 0.05; d = 0.34) scores compared to older athletes (i.e., > 25 years). Individual-sport athletes reported higher ESS (p < 0.05; d = 0.37) and lower QOL (p < 0.01; d = 0.51) scores compared to team-sport athletes. Higher DASS scores were associated with higher PSQI (t = 3.68; β = 0.3) and ISI (t = 4.78; β = 0.36) scores. Lower physical health (i.e., sub-scale of WHOQOL) was associated with higher DASS (t = -5.01; β = -0.42) and ISI (t = -8.02; β = -0.61) scores. Higher PSQI scores contributed to lower WHOQOL scores (t = -4.81; β = -0.41). In summary, the current study highlights reciprocal relationships between low sleep quality, low mental health and low QOL. Elite athletes (especially sub-groups of female, individual, and young athletes) showed a low sleep quality, potentially affecting their physical and psychological health and QOL.

## LIST OF ABBREVIATIONS

°C: Celsius

d: Cohen’s effect size

DASS: depression, anxiety and stress score

EDS: excessive daytime sleepiness

ESS: Epworth sleepiness scale

hh:mm: hour:minute

ISI: insomnia severity index

min: minute

N°: number

PMS: percent of missed sleep

PSQI: Pittsburgh Sleep Quality Index

QOL: quality of life

R^2^: the proportion of variance in the dependent variable explained by independent variables

sr^2^: to indicate the unique contribution (in percentage) of each independent variable within the model

SE: sleep efficiency

SOL: sleep onset latency

TIB: time in bed

TST: total sleep time

WHOQOL: world health organization quality of life

WHOQOL-BREF: world health organization quality of life brief version yrs.: years

## KEY POINTS

– Bidirectional relationships were found between low sleep quality, low mental health, and low quality of life in elite athletes.
– Subgroups of females, young, unsatisfied with their career, and individual sports athletes reported an even lower sleep quality compared to male, old, satisfied with their career, and team sports athletes.
– Longer career duration is associated with lower quality of life in elite athletes.

## INTRODUCTION

Elite athletes are exposed to high training loads to achieve physical and psychological adaptation to highly demanding competitions [1]. A high level of physical and psychological stress requires an adequate amount of recovery to maximise adaptations [2]. An imbalance between high training loads and inadequate recovery may result in a compromised physical and mental health [1]. Importantly, sleep has always been recognised as the most important recovery strategy by elite athletes [2]. Because of the sport-induced higher physiological and psychological strain, elite athletes may require longer sleep duration than the recommended 7–9 hours for healthy adults [3]. Indeed, joint consensus suggested that elite athletes may require 9–10 hours to optimise athletic performance and to preserve mental and physical health [1, 4–6].

Despite the well-acknowledged importance of obtaining an adequate amount of sleep for mental and physical health, elite athletes often present a low sleep quality and quantity, with female, individual-sports and older athletes being particularly more vulnerable to sleep disruptions [1, 2, 6, 7]. Several sport related (e.g., high training loads, night competitions, trans-meridian travel, unfamiliar sleep surrounding, and/or sponsors commitments), and non-sport related (e.g., chronotype, social/family commitments, work/study commitments and/or individual characteristics) factors may contribute to this suboptimal sleep, systematically reviewed elsewhere [1, 8–11]. Moreover, various lifestyle behaviours (e.g., caffeine, alcoholic and sugar-sweetened beverages, late and/or long daytime naps, nocturnal bright light exposure, late and/or large night meals) have been identified as potential sleep disrupters [12–16]. Acute exposure to a sleep disrupter might be tolerated; however, when disrupted sleep becomes the rule rather than the exception, repercussions on sporting performances, mental, and/or physical health might be expected [2]. In the general population, a reciprocal relationship between lower sleep quality and lower mental health has been described [17]. Further, chronic sleep restriction has been (i) linked to the onset and amplification of physical pain, and (ii) shown as affecting psychological health through compromising an optimistic outlook [18]. Indeed, insufficient and irregular sleep patterns have been shown to contribute to the onset, recurrence, and maintenance of mental health difficulties in non-athletic individuals [17]. In an international sample of > 3300 students, a better sleep quality was directly associated with better quality of life (QOL) [19]. This relationship was indirectly mediated by depression score [19], highlighting the interconnection between sleep, mental and physical health and QOL. However, little is known about the relationship between sleep quality, mental health and QOL in highly trained athletes.

Understanding the relationship between sleep and mental and physical health in elite athletes will help individualise training loads and recovery, allowing specialists to provide timely interventions and prevent injuries, overtraining, and mental burnout. Furthermore, enhancing sleep quality will, in turn, support emotional well-being and life satisfaction, thereby optimising athletes’ performance and fostering a long-lasting career. Therefore, this study aimed to (i) assess the relationship between sleep quality, QOL and psychological health in a sample of elite athletes; (ii) characterise their sleep, with between-subgroup analysis; and (iii) identify the behavioural causes contributing to the lower sleep quality. Based on the abovementioned considerations, we expect (i) a reciprocal relationship between sleep quality, QOL and psychological health; (ii) a low sleep quality in this sample, especially in female and individual athletes; and (iii) that these relationships will be influenced by behavioural factors related to sleep hygiene.

## MATERIALS AND METHODS

### Participants

Informed consent was provided by participants under ethical approval from the IRB Ethical Board of the ‘’Farhat Hached Hospital’’, Sousse, Tunisia (IORG 0007439, ERC 01/03/2024) in the spirit of the Helsinki Declaration. Data were processed anonymously and according to the guidelines of the “General Data Protection Regulation” (gdpr-info.eu). Eligibility criteria to be included in this study were: (i) ≥ 18 years of age; (ii) classified by the correspondent organization (Ministry of sports, national program of elite sport development) as an elite athlete (individual or team sport) of both sexes; and (iii) not suffering/have not suffered a major injury in the previous month.

### Power analysis

A total of 118 elite athletes took the survey based on a priori calculated sample size using the G*Power software (University of Düsseldorf, Germany). The study was designed to investigate the relationship between sleep quality and QOL in Tunisian elite athletes. Therefore, the F family test was chosen, with linear multiple regression as the statistical test. The effect size (f^2^ = 0.408) was calculated based on an earlier study using a similar paradigm Romdhani et al. [5]. Alpha was set at p = 0.05, beta was set at 0.01 to achieve a higher level of confidence, the number of tested predictors was 12, and the number of total predictors was 24. The G*Power software indicated a minimum of 97 participants to achieve a power of 0.99.

### Procedure

The study is part of a Tunisian national project aiming to enhance awareness about the importance of appropriate sleep hygiene, highlighting the interaction between sleep and athletic performance. Several awareness sessions were organised by a team of sleep and athletic performance scientists. The awareness sessions were presented by the first author after a discussion with the team members to adjust the content. They consist of a 20-minute PowerPoint ® presentation focusing on evidence-based knowledge. It highlighted the importance of adequate quality/quantity of sleep for recovery and optimal performance, as well as sleep hygiene aspects (discussed in depth in the discussion section) that may interfere with sleep. The presentation was followed by a further 10-minute question-andanswer discussion. These sessions were held in collaboration with the Tunisian Ministry of Sports and Youth and the national federations of: judo, karate, rowing, rugby, taekwondo, tennis, track and field, volleyball and wushu kung-fu. Sessions were held with small groups (10–15 athletes per session) and scheduled at the same time of day (16h00–18h00), where the athletes answered the survey on their phones or personal computers.

### The survey

The survey consisted of six sections and was presented to athletes on a Google Form ® in Arabic. The full English version of the survey can be found within the Supplementary File.

#### Section 1: Preamble & demographic questions

This section explained the aim of the study, invited athletes to confirm eligibility and provide consent to participate, encouraging them to respond as accurately as possible.

Demographic questions included sex, age, the sport discipline, the level of competition, and the highest sporting achievement(s). Participants were also asked if they were satisfied with their career so far (highly satisfied, satisfied, not satisfied, and not at all satisfied, subsequently grouped into satisfied and unsatisfied to facilitate binary comparison. For analysis purposes, subgroups were created: sex (male vs. female), age (≤ 25 yrs. vs. > 25 yrs.) based on the World Health Organisation classification of young people [20], level of competitiveness (world class vs. international) based on the taxonomy proposed by McKay et al. [21], sport discipline (individual vs. team), and satisfaction with their performance so far (satisfied vs. unsatisfied).

#### Section 2: Training questions

Participants were asked about their preferred time of day to train (hh: mm, regardless of the number of training sessions per day), the actual average number of training sessions per week (training frequency) and the mean duration of training sessions they were regularly performing.

#### Section 3: sleep quality, insomnia and daytime sleepiness

The validated Arabic version [22] of the Pittsburgh Sleep Quality Index (PSQI) assesses subjective sleep quality by answering 10 questions, with a score ranging from 0 to 21; a score between 5 and 7 indicates poor sleep quality, and a score of ≥ 8 indicates very poor sleep quality. The PSQI questionnaire also determines self-reported bed and wake-up times (hh: mm), total sleep time (TST, min), time in bed (TIB, min), sleep onset latency (SOL, min) and sleep efficiency (SE, %; TST/TIB × 100). An additional question [6] asked about the desired amount of sleep to feel fully rested (Desired TST), with the percentage of missed sleep (PMS, %) subsequently calculated according to the formula [100 – (TST/Desired TST) × 100].

The validated Arabic version [23] of the insomnia severity index (ISI) contains seven questions to assess the severity of insomnia symptoms. Total ISI score ranges from 0–28, indicative of moderate (15–21) or severe (22–28) clinical insomnia.

The validated Arabic version [24] of the Epworth sleepiness scale (ESS) was used to measure the subjective daytime sleepiness. The original questionnaire contains eight questions with an overall score ranging from 0 to 24. The ESS score was interpreted as follows: < 10: normal; 11-to-15: excessive-; and ≥ 16: severe- daytime sleepiness.

#### Section 4: The world health organization quality of life short-version (WHOQOL-BREF)

The validated Arabic version [25] of WHOQOL-BREF is an abbreviated version (i.e., 26 items) of the original WHOQOL-100. The WHOQOL-BREF assesses the QOL in four domains: physical, psychological, and environmental health, and social relationships. Respondents rate each item on a Likert scale, indicating their level of agreement or satisfaction. The responses are then used to compute scores for each domain, providing an overall picture of an individual’s QOL across these four dimensions. For statistical purposes, the WHOQOL overall score was used for the multiple regression analysis.

#### Section 5: Depression, anxiety and stress scale 21

The validated Arabic version [26] of DASS-21 is a self-report questionnaire used to assess the levels of depression, anxiety, and stress experienced by individuals. It consists of 21 items, with 7 items dedicated to each of the three constructs: depression, anxiety, and stress. Participants were asked to rate the extent to which they have experienced various emotional symptoms over the past week. For statistical purposes, the DASS overall score was retained for betweengroups and multiple regression analysis.

#### Section 6: Napping & Sleep hygiene questions

This section encompasses questions related to sleep hygiene given the fact that sleep quality might be affected by several aspects such as, but not limited to: napping, quantity and time of food and beverage, light-emitting devices exposure [12, 13, 16, 27, 28]. The reliability of these questions has been studied elsewhere [5, 29], ranging from good (r = 0.83) to very good (r = 0.97). Participants were asked about their napping behaviour [i.e., nap timing (hh: mm), duration (min) and frequency (N° per week)]. Nutrition-related questions included the number of meals and snacks (N° per day), and the last meal of the day’ time (hh: mm). Furthermore, participants were asked about how many sodas and alcoholic beverages they consumed (N° per week), how many caffeinated beverages they consumed, and how many cigarettes they eventually smoked (N° per day). Finally, participants were asked about their estimation of the duration (min per day) they spend on screens (e.g., TVs, tablets and smartphones).

### Statistical analysis

Statistical analysis was performed using SPSS (IBM Corp. IBM SPSS Statistics for Windows, Version 26. Armonk, NY: IBM Corp), and figures were created using GraphPad Prism 8 (GraphPad Software, San Diego, CA, USA). The Kolmogorov-Smirnov test confirmed the normal distribution of data; therefore, parametric tests were used. The independent sample t-test was used to compare between subgroups of sex, competitive level, sport discipline, age, and satisfaction. The Cohen’s effect size (d) was subsequently calculated, qualitatively interpreted as small (d < 0.5), moderate (0.5 ≤ d < 0.8) and large (d ≥ 0.8), to assess the magnitude of the difference [30].

Multiple linear regression analyses described the relationships between dependent (PSQI, ISI, DASS and WHOQOL scores) and independent (sleep, training, health and nutrition-related) variables. All the independent variables were entered into the model using a stepwise method. We report the R-squared (R^2^; the proportion of variance in the dependent variable explained by independent variables) for the entire model as well as the semi-partial correlation coefficient squared (sr^2^) to indicate the unique contribution (in percentage) of each independent variable within the model. The multiple regression model was completed by further statistical mediation to quantify the indirect effect of independent variables on the dependent variables (i.e., PSQI, ISI, DASS and WHOQOL). The mediation analysis was conducted according to Hayes’ guidelines [31], using the PROCESS macro for SPSS. The magnitude of the mediation effect is expressed as a percentage according to the formula (indirect effect/total effect * 100). All values within the text, figures and tables are reported as mean ± standard deviation (SD), unless stated otherwise. Alpha was set at p < 0.05.

## RESULTS

### Participants

An overall sample of 118 elite athletes was included in the study (33% female, 20.1 ± 0.64 yrs., range: 18–37 yrs.), practising their respective sports for 9.6 ± 2.53 (range: 3–27) yrs., and training an average of 850 ± 36 (range: 420–1500) min · week^−1^. The participants practised judo (n = 14), karate (n = 10), rowing (n = 8), rugby (n = 20), tennis (n = 4), taekwondo (n = 9), track and field (n = 15), volleyball (n = 22), wrestling (n = 9) and wushu kung-fu (n = 7). Therefore, they were divided into sub-groups of males (n = 79) and females (n = 39); ≤ 25 yrs. (n = 102) and > 25 yrs. (n = 16); world class (n = 50) and international (n = 68); individual- (n = 76) and team-sports (n = 42) athletes; and satisfied (n = 74) and unsatisfied (n = 44) with their sport performance achievements so far.

### Sleep quality, insomnia severity and daytime sleepiness

**FIG. 1:**
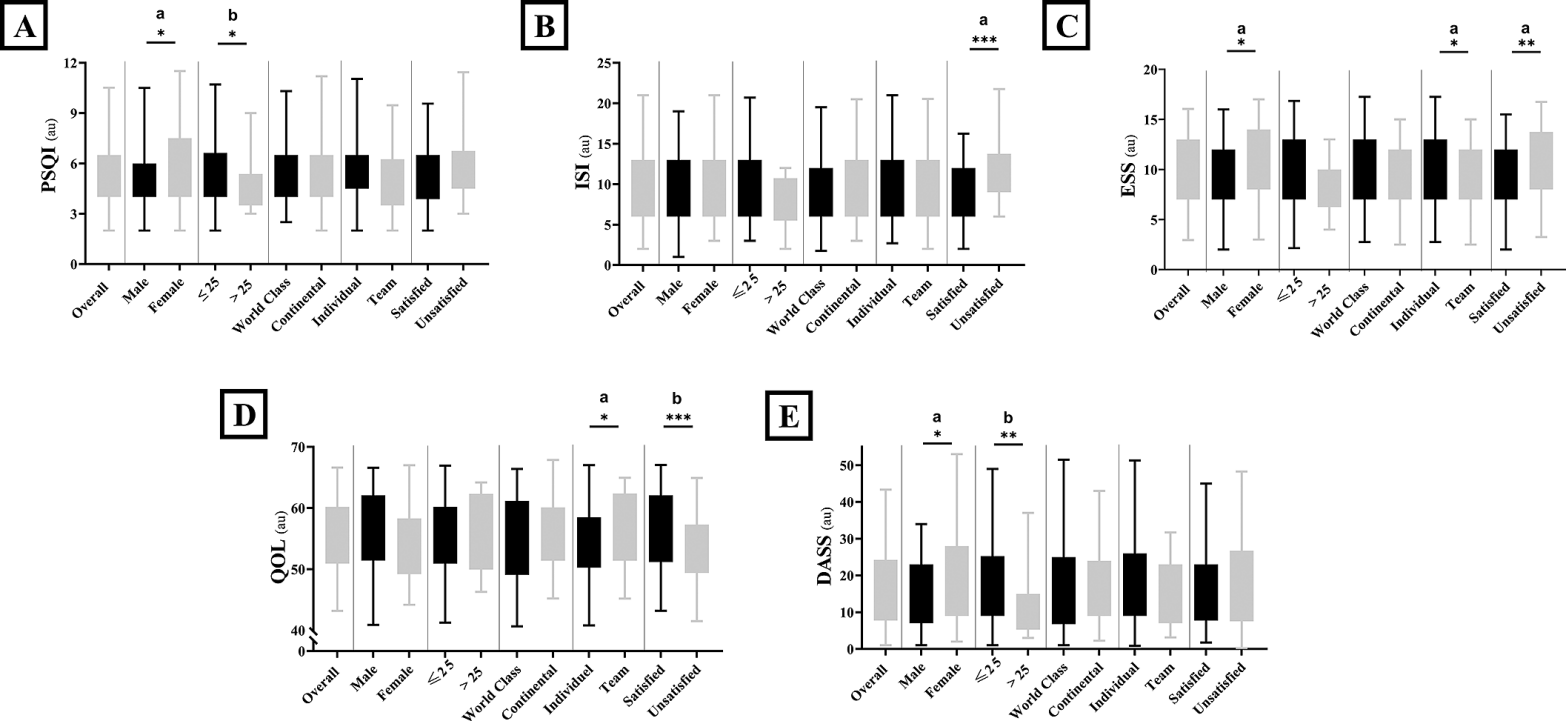
Box and whiskers of the Pittsburgh sleep quality index (a: PSQI), insomnia severity index (b: ISI), Epworth sleepiness scale (c: PSQI), the quality of life (d: QOL) and the depression, anxiety, stress scale (e: DASS) for the overall sample (n = 118) and for subgroups; male (n = 79) and female (n = 39) athletes; ≤ 25 years (n = 102) and > 25 years (n = 16); World class elite (n = 50) and international elite (n = 68); individual (n = 76) and team sport (n = 42) athletes; satisfied (n = 74) and unsatisfied (n = 44) with their achieved performance so far. *, **and *** means significant within group difference at p < 0.05, p < 0.01 and p < 0.001, respectively, with a, b and c presenting; small, medium and large Cohen’s effect size, respectively. Data are presented as mean and 95% confidence interval and the difference between subgroups was assessed by the independent sample t test.

Of the overall sample, 47% and 15% reported poor and very poor sleep quality, respectively; 14% and 3% had moderate and severe insomnia; and 48% and 5% reported excessive and pathological daytime sleepiness, respectively. PSQI (Figure 1a), ISI (Figure 1b) and ESS (Figure 1c) values for the overall sample and between-subgroup comparison are displayed in Figure 1. The proportions of good, poor and very poor sleep quality; normal, moderate and severe insomnia; normal, excessive and pathological daytime sleepiness for the overall sample and for each subgroup are presented in Table 1.

**TABLE 1:** Distribution (in percentage) of the selected sleep quality parameters for the overall sample and between subgroups.

Parameters (unit)	Value	Overall (n=118)	Sex		Age	

			Male (n=79)	Female (n=39)	< 25 yrs. (n=102)	> 25 yrs. (n=16)
Proportion of good sleep quality (%)	PSQI score < 5	38.8	39.74	36.84	34.65	75
Proportion of low sleep quality (%)	PSQI score ≥ 5	46.61	48.72	39.47	49.5	12.5
Proportion of very low sleep quality (%)	PSQI score ≥ 8	15.25	11.54	23.68	15.84	12.5
Proportion of no EDS (%)	ESS score < 10	48.31	56.41	36.84	47.06	68.75
Proportion of moderate EDS (%)	ESS score ≤ 15	45.76	34.62	44.74	38.24	31.25
Proportion of severe EDS (%)	ESS score ≥ 16	5.08	8.97	18.42	13.73	0
Proportion of no insomnia (%)	ISI score < 15	83.9	84.62	78.95	81.37	87.5
Proportion of moderate insomnia (%)	ISI score ≥ 15	13.56	12.82	21.05	16.67	12.5
Proportion of severe insomnia (%)	ISI score ≥ 22	2.54	2.56	0	1.96	0
Proportion of short TST (%)	TST < 7 hours	29.66	30.77	28.95	32.35	12.5
Proportion of least normal TST (%)	TST 7 hours	29.66	25.64	36.84	29.41	31.25
Proportion of normal TST (%)	TST 7–9 hours	38.14	42.31	31.58	37.25	43.75
Proportion of long TST (%)	TST > 9 hours	2.54	1.28	2.63	0.98	12.5
Proportion of normal SOL (%)	SOL ≤ 30 min	83.9	92.31	68.42	84.31	75
Proportion of long SOL (%)	SOL > 30 min	16.1	7.69	31.58	15.69	25
Proportion of normal SE (%)	SE < 85%	70.34	70.51	71.05	67.65	87.5
Proportion of low SE (%)	SE ≥ 85%	29.66	29.49	28.95	32.35	12.5


**Parameters (unit)**	**Level**		**Discipline**		**Satisfaction**	

	**World class (n=50)**	**International (n=68)**	**Individual (n=76)**	**Team (n=42)**	**Satisfied (n=74)**	**Unsatisfied (n=44)**

Proportion of good sleep quality (%)	37.74	41.27	31.58	50	39.73	39.53
Proportion of low sleep quality (%)	49.06	42.86	50	35.71	46.58	41.86
Proportion of very low sleep quality (%)	13.21	15.87	18.42	14.29	13.7	18.6
Proportion of no EDS (%)	45.28	53.97	44.74	54.76	54.79	39.53
Proportion of moderate EDS (%)	39.62	38.10	36.84	42.86	35.62	44.19
Proportion of severe EDS (%)	15.09	9.52	18.42	2.38	9.59	16.28
Proportion of no insomnia (%)	83.02	84.13	80.26	85.71	80.82	86.05
Proportion of moderate insomnia (%)	15.09	14.29	17.11	11.90	17.81	11.63
Proportion of severe insomnia (%)	1.89	1.59	2.63	2.38	1.37	2.33
Proportion of short TST (%)	25.93	32.81	36.84	16.67	31.51	25.58
Proportion of least normal TST (%)	33.33	26.56	32.89	23.81	19.18	48.84
Proportion of normal TST (%)	40.74	37.50	30.26	52.38	47.95	23.26
Proportion of long TST (%)	0	3.13	0	7.14	1.36	0
Proportion of normal SOL (%)	79.63	87.5	85.53	80.95	89.04	76.74
Proportion of long SOL (%)	20.37	12.5	14.47	19.05	10.96	23.26
Proportion of normal SE (%)	75.93	67.19	75	64.29	67.12	76.74
Proportion of low SE (%)	24.07	32.81	25	35.71	32.88	23.26

ESS: Epworth Sleepiness Scale; EDS: excessive daytime sleepiness; ISI: Insomnia Severity Index; PSQI: Pittsburgh Sleep Quality Index; (%): percent; TST: Total Sleep Time; SE: Sleep Efficiency; SOL: Sleep Onset Latency and yrs.: years.

### Sleep behaviours

Of the overall sample, 30% reported sleeping less than 7 hours, 30% sleeping 7 hours, 38% sleeping between 7 and 9 hours, and 3% sleeping more than 9 hours. In addition, 30% of the athletes reported a SE lower than 85%, synonymous with low SE. 60% reported a SOL of 30 min or longer, indicative of longer than normal SOL. Table 2a reports the values of the selected parameters for the overall sample and the comparison between subgroups. The proportion of the selected sleep parameters for the overall sample and for each subgroup is presented in Table 1.

**TABLE 2.A:** mean ± SD (standard deviation) of sleep and napping parameters of the overall sample and between subgroups.

Parameters (unit)	Overall (n=118)		Sex		Age	

			Male (n=79)	Female (n=39)	≤ 25 yrs. (n=102)	> 25 yrs. (n=16)
Bedtime (hh:mm)	23:06 ± 1:01		23:09 ± 1:06	22:59 ± 0:46	23:03 ± 1:04	23:15 ± 0:45
Wake time (hh:mm)	7:21 ± 1:20		7:26 ± 1:22	7:09 ± 1:16	7:17 ± 1:22	7:42 ± 1:05
SOL (min)	29.31 ± 17.39		**26.52 ± 14.57**	34.97 ± 21.12^** b^	28.96 ± 16.82	31.56 ± 21.11
TIB (hh:mm)	8:15 ± 1:19		8:20 ± 1:20	8:08 ± 1:11	8:13 ± 1:19	8:25 ± 1:04
SE (%)	86.08 ± 8.11		85.84 ± 8.72	86.57 ± 6.79	85.72 ± 8.57	88.41 ± 3.51
TST (hh:mm)	7:05 ± 1:12		7:06 ± 1:15	7:03 ± 1:05	7:01 ± 1:14	7:28 ± 1:06
Desired TST (hh:mm)	9:07 ± 1:24		**8:57 ± 1:30**	9:28 ± 1:15^* b^	9:08 ± 1:27	9:04 ± 1:16
Missed sleep (%)	21.23 ± 13.26		**19.49 ± 13.42**	24.57 ± 12.36^* b^	21.91 ± 13.34	16.86 ± 21.24
Nap frequency (N° · week^−1^)	3.55 ± 2.02		3.34 ± 1.75	3.97 ± 2.46	3.48 ± 2.03	4.08 ± 1.92
Nap duration (min)	95.01 ± 46.14		95.29 ± 47.25	94.43 ± 43.98	**98.92 ± 46.62**	63.75 ± 24.87^*c^
Nap timing (hh:mm)	13:43 ± 0:51		**13:39 ± 0:49**	14:08 ± 0:51^* c^	**13:44 ± 0:52**	14:24 ± 0:30^*c^
Nap per day (min · day^−1^)	48.08 ± 38.95		**43.2 ± 32.92**	58.57 ± 48.39^* b^	**49.85 ± 40.67**	34.11 ± 15.51^*b^


**Parameters (unit)**	**Level**		**Discipline**		**Satisfaction**	

	**World class (n=50)**	**International (n=68)**	**Individual (n=76)**	**Team (n=42)**	**Satisfied (n=74)**	**Unsatisfied (n=44)**

Bedtime (hh:mm)	23:06 ± 0:53	23:05 ± 1:07	23:01 ± 1:03	23:12 ± 0:59	23:00 ± 0:59	23:14 ± 1:05
Wake time (hh:mm)	7:28 ± 1:13	7:15 ± 1:25	**7:03 ± 1:15**	7:53 ± 1:20^***a^	7:29 ± 1:19	7:07 ± 1:21
SOL (min)	27.61 ± 12.09	30.57 ± 20.43	28.61 ± 16.89	30.61 ± 18.38	28.16 ± 18.38	31.25 ± 15.59
TIB (hh:mm)	8:20 ± 1:03	8:15 ± 1:26	**8:02 ± 1:10**	8:39 ± 1:26^**a^	**8:24 ± 1:18**	7:59 ± 1:16^*b^
SE (%)	**87.46 ± 5.45**	85.07 ± 9.52^*a^	85.61 ± 9.13	86.94 ± 5.83	86.08 ± 7.43	86.09 ± 9.23
TST (hh:mm)	7:16 ± 0:54	6:56 ± 1:23	**6:52 ± 1:09**	7:30 ± 1:16^**b^	**7:14 ± 1:12**	6:52 ± 1:11^*b^
Desired TST (hh:mm)	9:11 ± 1:27	9:04 ± 1:22	**8:53 ± 1:26**	9:31 ± 1:17^**b^	9:12 ± 1:19	8:59 ± 1:32
Missed sleep (%)	19.41 ± 12.89	22.56 ± 13.47	21.74 ± 12.43	20.29 ± 14.76	20.67 ± 13.51	22.16 ± 12.95
Nap frequency (N° · week^−1^)	3.91 ± 1.99	3.27 ± 2.01	**3.93 ± 2.07**	2.78 ± 1.69^***b^	3.66 ± 1.87	3.35 ± 2.25
Nap duration (min)	94.09 ± 49.25	95.69 ± 43.85	97.1 ± 47.61	90.83 ± 42.98	96.56 ± 46.45	92.38 ± 45.72
Nap timing (hh:mm)	13:44 ± 1:01	13:52 ± 0.43	13:44 ± 0:52	13:58 ± 0:50	13:45 ± 0:58	13:54 ± 0:39
Nap per day (min · day^−1^)	52.61 ± 38.26	44.81 ± 39.41	**54.91 ± 43.57**	34.06 ± 21.54^**b^	48.42 ± 36.76	47.49 ± 42.98

Between groups difference was assessed with an independent sample t-test. *, ** and *** mean significant difference between groups at p < 0.05, p < 0.01 and p < 0.001, respectively. Significant comparisons are highlighted in bold font, with a, b and c presenting; small, medium and large Cohen’s effect size, respectively.

hh:mm: hours: minutes; min: minutes; N°: number; TIB: Time in Bed; TOD: time of day; TST: Total Sleep Time; SE: Sleep Efficiency; SOL: Sleep Onset Latency and yrs.: years

### WHOQOL and DASS

The values of WHOQOL subscales are presented in Table 2b, and Figure 1 presents the overall score of WHOQOL (Figure 1d) and DASS (Figure 1e) for the overall sample and the comparison between subgroups.

**TABLE 2.B:** Mean ± SD (standard deviation) of training, nutrition, sleep hygiene-related, and quality of life parameters of the overall sample and between subgroups.

Parameters (unit)	Overall (n=118)		Sex		Age	

			Male (n=79)	Female (n=39)	≤ 25 yrs. (n=102)	> 25 yrs. (n=16)
Training TOD (hh:mm)	14:18 ± 4:37		14:36 ± 4:38	13:43 ± 4:37	14:32 ± 4:42	12:54 ± 3:54
Training duration (min · week^−1^)	848 ± 348		848 ± 380	848 ± 277	**818 ± 359**	1040 ± 180^***c^
Caen (au)	52.31 ± 7.67		51.81 ± 8.53	53.31 ± 5.36	52.61 ± 8.04	50.38 ± 4.33
Meals (N° · day^−1^)	3.27 ± 0.72		**3.41 ± 0.74**	3 ± 0.56^*** a^	3.31 ± 0.74	3.06 ± 0.44
Snacks (N° · day^−1^)	1.64 ± 1.08		1.73 ± 1.19	1.46 ± 0.79	**1.75 ± 1.09**	0.94 ± 0.68^***c^
Total meals (N° · day^−1^)	4.91 ± 1.45		**5.13 ± 1.58**	4.46 ± 1.02^** b^	**5.05 ± 1.47**	4 ± 0.89^***c^
Last meal time (hh:mm)	19:32 ± 4:25		19:08 ± 5:11	20:22 ± 1:53	19:23 ± 4:43	20:24 ± 1:17
Caffeine (N° · day^−1^)	1.17 ± 0.87		1.24 ± 0.96	1.03 ± 0.64	1.1 ± 0.88	1.56 ± 0.72
Caffeine TOD (hh:mm)	10:00 ± 4:16		10:23 ± 4:35	9:16 ± 3:33	10:09 ± 4:30	9:04 ± 2:26
Alcohol (N° · week^−1^)	0.37 ± 1.65		0.52 ± 1.98	0.08 ± 0.48	0.33 ± 1.84	0.63 ± 2.51
Soda (N° · week^−1^)	2.35 ± 2.86		2.34 ± 2.51	2.38 ± 3.49	**2.56 ± 2.96**	1.06 ± 1.73^*b^
Cigarette (N° · day^−1^)	0.26 ± 1.92		0.39 ± 1.57	0 ± 0	0.21 ± 1.2	0.63 ± 2.51
Screen duration (min · day^−1^)	92.67 ± 77.42		100.5 ± 82.6	77.05 ± 63.5	91.71 ± 71.82	98.75 ± 48.14
Physical Health (au)	14.12 ± 1.98		14.31 ± 2.04	13.75 ± 1.81	14.07 ± 2.02	14.46 ± 1.68
Psychological Health (au)	14.21 ± 2.32		14.26 ± 2.51	14.11 ± 1.93	14.09 ± 2.42	14.92 ± 1.41
Social interaction (au)	14 ± 3.11		**14.36 ± 3.31**	13.27 ± 2.59^* b^	13.96 ± 3.23	14.27 ± 2.26
Environmental health (au)	12.51 ± 2.35		12.46 ± 2.44	12.6 ± 2.21	12.41 ± 2.32	13.13 ± 2.54


**Parameters (unit)**	**Level**		**Discipline**		**Satisfaction**	

	**World class (n=50)**	**International (n=68)**	**Individual (n=76)**	**Team (n=42)**	**Satisfied (n=74)**	**Unsatisfied (n=44)**

Training TOD (hh:mm)	**13:18 ± 4:32**	15:08 ± 4:34^**b^	14:08 ± 4:50	14:35 ± 4:16	**15:02 ± 4:15**	13:01 ± 5:01^***a^
Training duration (min · week^−1^)	**757 ± 347**	957 ± 320^***b^	885 ± 377	782 ± 281	**757 ± 303**	1002 ± 368^***b^
Caen (au)	51.64 ± 6.35	52.79 ± 8.52	52.55 ± 6.96	51.86 ± 8.87	**53.31 ± 6.17**	50.61 ± 9.52^*a^
Meals (N° · day^−1^)	3.16 ± 0.58	3.35 ± 0.78	3.24 ± 0.67	3.33 ± 0.78	3.35 ± 0.67	3.14 ± 0.76
Snacks (N° · day^−1^)	**1.34 ± 0.77**	1.87 ± 1.22^**a^	1.62 ± 1.01	1.69 ± 1.19	1.55 ± 1.14	1.81 ± 0.95
Total meals (N° · day^−1^)	**4.51 ± 1.03**	5.22 ± 1.63^**b^	4.85 ± 1.39	5.02 ± 1.56	4.91 ± 1.49	4.93 ± 1.41
Last meal time (hh:mm)	19:54 ± 3:04	19:15 ± 5:11	19:32 ± 3:52	19:31 ± 5:20	19:26 ± 4:13	19:42 ± 4:48
Caffeine (N° · day^−1^)	1.28 ± 0.75	1.08 ± 0.95	1.25 ± 0.94	1.02 ± 0.73	**1.03 ± 0.79**	1.4 ± 0.95^**a^
Caffeine TOD (hh:mm)	10:32 ± 3:58	9:33 ± 4:29	10:29 ± 4:22	9:06 ± 4:00	9:29 ± 4:13	10:46 ± 4:16
Alcohol (N° · week^−1^)	0.49 ± 1.98	0.28 ± 1.36	0.31 ± 1.52	0.48 ± 1.86	0.31 ± 1.43	0.51 ± 1.99
Soda (N° · week^−1^)	**1.74 ± 2.25**	2.82 ± 3.19^**b^	2.15 ± 2.61	2.71 ± 3.27	2.31 ± 2.84	2.43 ± 2.94
Cigarette (N° · day^−1^)	0.37 ± 1.57	0.18 ± 1.03	0.11 ± 0.71	0.55 ± 1.91	0.28 ± 1.43	0.23 ± 1.02
Screen duration (min · day^−1^)	97.65 ± 86.92	89.03 ± 69.84	91.36 ± 73.72	94.98 ± 84.04	96.96 ± 80.34	85.41 ± 72.11
Physical Health (au)	14.34 ± 1.75	13.97 ± 2.12	14.16 ± 2.07	14.05 ± 1.82	14.27 ± 1.88	13.88 ± 2.12
Psychological Health (au)	14.58 ± 2.17	13.93 ± 2.41	13.97 ± 2.49	14.64 ± 1.92	**14.67 ± 2.11**	13.43 ± 2.49^***b^
Social interaction (au)	13.85 ± 3.29	14.11 ± 3.01	13.82 ± 2.88	14.33 ± 3.51	**14.37 ± 3.16**	13.38 ± 2.97^*a^
Environmental health (au)	12.59 ± 2.21	12.45 ± 2.47	**11.99 ± 2.19**	13.44 ± 2.37^***b^	**12.99 ± 2.35**	11.69 ± 2.16^***b^

Between groups difference was assessed with an independent sample t-test. *, ** and *** mean significant difference between groups at p < 0.05, p < 0.01 and p < 0.001, respectively. Significant comparisons are highlighted in bold font, with a, b and c presenting; small, medium and large Cohen’s effect size, respectively.

au: arbitrary unit; hh:mm: hours: minutes; min: minutes; N°: number; TOD: time of day; and yrs.: years.

### Multiple regression and mediation

**FIG. 2:**
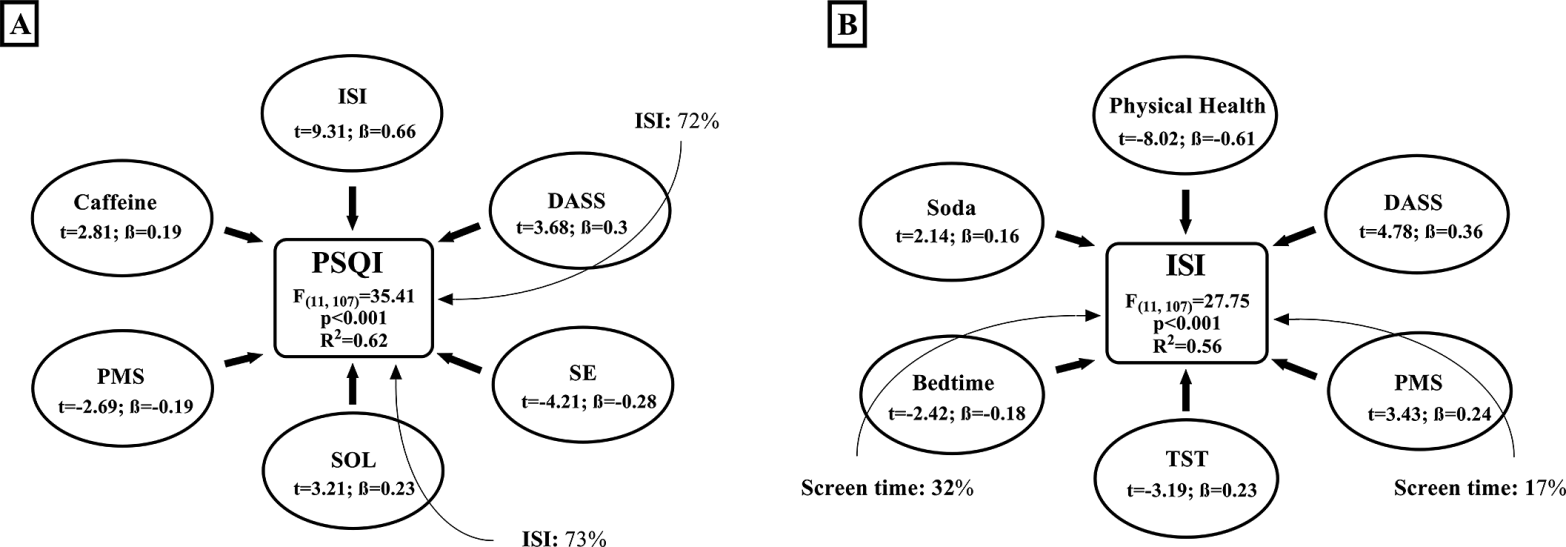
Multiple regression and mediation for the: Pittsburgh sleep quality index (PSQI, Figure 2a). Other independent variables significantly contributing to the model include; total sleep time: t = -2.98, ß = -0.21, p < 0.001; psychological health: t = -2.59, ß = -0.18, p < 0.01; and soda beverages: t = 2.37, ß = 0.17, p < 0.05. Insomnia severity index (ISI, Figure 2b). Other independent variables significantly contributing to the model include; sleep onset latency: t = 2.31, ß = 0.15, p < 0.05; training time of day: t = 2.28, ß = 0.15, p < 0.05; and screen duration: t = 1.98, ß = 0.14, p < 0.05. The stepwise method was used to determine the independent variables significantly contributing to the model. The bold arrows present the direct effect (multiple regression) of independent variables on the dependent variable. The magnitude of the association between independent and dependent variable is expressed as the semi-partial correlation coefficient squared (i.e., in percentages; the unique contribution of each independent variable within the model). The light arrows present the indirect effects (mediation) of independent variables on the dependent variable. The magnitude of the mediation effect is expressed as percentages according to the formula (indirect effect/total effect * 100). DASS: depression, anxiety and stress scale; ISI: insomnia severity index; PMS: percent of missed sleep; SE: sleep efficiency; SOL: sleep onset latency; TST: total sleep time.

**FIG. 3:**
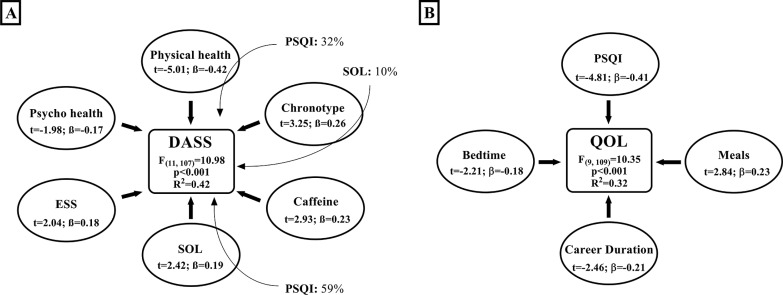
Multiple regression and mediation for: Depression, anxiety, stress scale (DASS, Figure 3a). Other independent variables significantly contributing to the model include; nap timing: t = -2.28, ß = -0.18, p < 0.01 and social interaction: t = 2.15, ß = 0.18, p < 0.01. Quality of life (QOL, Figure 3b). The stepwise method was used to determine the independent variables significantly contributing to the model. The bold arrows present the direct effect (multiple regression) of independent variables on the dependent variable. The magnitude of the association between independent and dependent variable is expressed as the semi-partial correlation coefficient squared (i.e., in percentages; the unique contribution of each independent variable within the model). The light arrows present the indirect effects (mediation) of independent variables on the dependent variable. The magnitude of the mediation effect is expressed as percentages according to the formula (indirect effect/total effect * 100). ESS: Epworth sleepiness scale; PSQI: Pittsburgh sleep quality index; SOL: sleep onset latency.

The multiple regression analysis identified an interaction between sleep quality, negative emotions and the QOL (Figure 2a). Further, insomnia severity was associated with QOL, negative emotions and sleep hygiene parameters (Figure 2b). Figure 3A presents the association between higher daytime sleepiness, sleep hygiene, negative emotions and QOL. Besides, better QOL was associated with better sleep quality, which is shown in Figure 3b.

## DISCUSSION

The study aimed to investigate the relationship between sleep quality, psychological health and the quality of life (QOL) in a sample of 118 elite athletes, with between-subgroup comparison. The main finding was that sleep quality, psychological health and QOL were reciprocally associated, supporting our first hypothesis. A high proportion of the assessed elite athletes reported low sleep quality (62%), high daytime sleepiness (51%) and insomnia severity (16%), and less than optimal sleep duration for elite athletes (60%). Subgroups of females, younger, individual-sports and unsatisfied athletes reported lower sleep quality and lower QOL compared to male, older, team-sports and satisfied athletes, respectively, partly accepting our second hypothesis. The current findings may aid in early identification of high-risk subgroups of elite athletes that are more vulnerable to sleep complaints, potentially affecting their physical and mental health, as well as overall well-being. Furthermore, nutritional (i.e., caffeine and soda consumption) and behavioural (i.e., late bedtime and screen time duration) aspects of sleep hygiene were associated with lower sleep quality and higher insomnia severity, aligning with our third hypothesis.

### Sleep characteristics of elite athletes

Athletes reported sleeping for 07h05min ± 01h12min per night, aligning with earlier results from systematic reviews [8, 11, 32]. In fact, ~ 60% of the current sample reported obtaining the least recommended, or less, TST [3] per night for healthy adults (Table 2a), which is believed to be below the recommended TST for elite athletes [1, 4]. Although specific recommendations for elite athletes are lacking, consensus statements encourage athletes to obtain 9–10 hours of sleep per night to allow recovery from the physical and psychological strain of high-level competition [1, 5, 10]. Therefore, it is fair to assume that 60% of the current sample were achieving suboptimal TST. Moreover, the current sample of elite athletes reported a SE of 86 ± 8%, with 30% of the sample reporting a SE < 85%, indicative of low SE, aligning with earlier reports [8, 11, 33]. Furthermore, the current sample wanted to obtain 114 ± 81 min of extra sleep, with only 16 (13%) athletes obtaining the exact amount of sleep they want to have. Similarly, an earlier study reported an actual TST of 6.7 ± 0.8-h and athletes want to obtain 96 ± 60-min of extra sleep [6]. Subsequently, 51% reported moderate-to-severe excessive daytime sleepiness (EDS) and 16% reported moderate-to-severe insomnia. Importantly, EDS and moderate-to-severe insomnia are associated with increased risk of sportrelated concussions [34], indicating that a high proportion of the current sample are at a high risk of such injury.

Strikingly, 62% of the sample reported poor or very poor sleep quality, which is higher than reported by earlier studies: 52% by Halson et al. [2] for Australian elite athletes, and 41% by Knufinke et al. [35] for Dutch elite athletes. This might be explained by the fact that this study took place during September, and the questionnaires used refer to the previous month. Actually, the average temperature in Tunisia during August days is high (> 40 °C) and can still be high even during the night (> 30 °C). Therefore, the lower sleep quality among Tunisians, compared to Australian and Dutch athletes [2, 35], might be time-of-year related, due to the environmental factors, rather than a chronic disrupted sleep. Although 65% of the current sample did have air conditioning in their rooms (data not shown), to what extent this might influence the current finding is unclear.

### Multiple regression analysis

The results of the multiple regression analysis showed strong correlations between sleep quality, insomnia severity, negative emotions and QOL; the latter were also affected/mediated by sleep hygiene parameters (e.g., caffeine and soda consumption, late bedtime, and screen use duration).

Lower sleep quality was most associated with higher insomnia severity and every 0.26 increase in ISI score was associated with a one-point increase in PSQI score. Further, insomnia severity had an indirect negative impact on sleep quality through longer SOL (Figure 2a). This finding confirms that difficulties falling asleep is the most common sleep complaint among elite athletes, with presleep cognitive arousal as the main underlying mechanism [8]. In fact, negative emotions may increase pre-sleep cognitive arousal through rumination and the imagination/planification of different possible scenarios in sport events, in particular, or sport career in general. The current results showed that higher negative emotions were associated with lower sleep quality and higher insomnia severity (Figure 2a, 2b). In elite athletes, even acute sleep restriction (one night of 4h30 time in bed) has been shown to increase negative emotions and depressed mood the next day [36]. Furthermore, a 2021 meta-analysis highlighted that improving sleep quality in the general population was associated with improved mental health (i.e., lower depression, anxiety and stress) in a doseresponse manner [17].

Elite athletes mostly rely on sleep to recover from the physical and psychological strain imposed by training/competing at high levels [2]. Longer TST and lower PMS were associated with better sleep quality and lower insomnia severity, supporting the common belief that athletes require longer TST than the recommended sleep duration (i.e., 7–9 hours) for the general population [1, 6]. It has been reported that sleep extension enhances physical and executive functions [4, 32, 37] and athletes compensate for the lower sleep quality by increasing both sleep duration and daytime naps [5]. Acute sleep disruption increases (i) muscular and systemic inflammation [36, 38] and (ii) pain sensitivity through alterations in neuroendocrine function and the modulation of neurotransmitters [38]. Chronic sleep restriction subsequently contributes to the onset and the amplification of pain and lowers the subjective health perception [18]. Therefore, without an adequate amount of sleep, athletes will permanently feel unrested, potentially leading to an inferior subjective rate of physical and psychological health. When this period of intensive physical and psychological strain becomes longer, it will be accompanied by a stronger feeling of permanent unrest. This could be consolidated by the current results, since a longer sports career was associated with lower QOL (Figure 3b). Moreover, lower physical health was associated with higher insomnia and negative emotions (Figure 2b & 3a), which in turn lowered sleep quality (Figure 2a) while the lower sleep quality contributed the most to the lower QOL (Figure 3b). Interestingly, the current findings highlight a reciprocal interaction between QOL and sleep quality, encouraging further investigation to assess a causal relationship and elucidate the underlying mechanisms.

Late chronotype was associated with a higher DASS score, and this relationship was mediated by longer SOL. In the general population, early diurnal preference was associated with 23% lower risk of depression [39], and late chronotype was associated with a wide range of sleep complaints (i.e., longer SOL, higher insomnia symptoms, higher nightmares’ prevalence, and less refreshing sleep), implying that late sleepers are bad sleepers [40]. Actually, early bedtime (e.g., 22h00–23h00) coincides with the phase range of circadian promotion of sleep, which properly aligns the sleep phase with the human internal circadian clock [15, 41]. This reinforces our results since early bedtime was associated with a better QOL (Figure 3b). However, a common behaviour contributing to lower sleep quality is lying in bed while using smartphones to chat, play, or watch videos/ movies. As a matter of fact, this behaviour may have the greatest impact on athletes’ sleep since longer SOL directly contributed to higher PSQI, ISI, and DASS scores. Besides, the amount of time spent in front of screens was associated with higher insomnia directly and indirectly through later bedtime and higher PMS. In controlled laboratory settings, exposure to electronic light-emitting devices increases arousal, reduces sleep propensity and delays the circadian phase by up to 90 min [41]. Conversely, the removal of electronic devices for two consecutive days did not affect sleep quality and quantity, but athletes overestimated the subjective TST and underestimated SOL [28]. Importantly, our study was based on subjective rates of sleep parameters; therefore, it is possible that athletes overestimated the impact of light exposure on their sleep. Therefore, the effect of screens/bright light exposure during the evening on subsequent night’s sleep quality and quantity should not be neglected and requires further studies.

### Sex-based difference

TST was not different between female and male athletes. However, female athletes reported longer SOL, and wanted to have more sleep, resulting in higher PMS (Table 2a); the latter may explain their higher PSQI and ESS scores (Figure 1) and the longer daytime naps compared to males. Moreover, the females’ group had a higher proportion (24%) of very low sleep quality (i.e., PSQI score ≥ 8) compared to the males (12%). In general, females have a shorter circadian period, marked by an earlier timing of circadian rhythms of melatonin and body temperature [42]. This indicates that even when male and female athletes sleep at the same time, females are sleeping at a later biological time compared to males [43], which could explain the lower sleep quality perception [42]. Further, female athletes reported higher negative emotions scores (i.e., DASS) and lower physical and mental health scores (Figure 1, Table 2b), which were mutually associated with lower sleep quality (Figures 2, 3). Similarly, a higher prevalence of psychopathologies (anxiety, depression and self-harming behaviours) was reported among female compared to male French elite athletes [44]. Here, it appears that female athletes are at a higher risk of developing low sleep quality-induced psychological difficulties. This vulnerability can be attributed to various factors, including hormonal influences, shorter circadian period, psychological sensibility and temperature variations [43].

### Age-based difference

The younger group of athletes reported higher PSQI and DASS scores (both with medium effect size). Moreover, 65.3% of the younger group reported low or very low sleep quality compared to only 25% of the older group. Although the difference was not statistically significant, the older group reported sleeping 25 minutes longer than the younger group. One plausible explanation from the current data is that the younger group reported longer daytime naps, probably to compensate for the low sleep quality and/or the short sleep duration. Concerning daytime napping, the longer is not always the better, since too long daytime naps depress homeostatic sleep drive buildup and may disturb nocturnal sleep [2]. Indeed, a 20-minute nap enhanced cognitive and physical performances more than a 90-minute nap when the athletes obtained 8 hours of time in bed [45], and this effect was even higher when the athletes obtained a moderate dose of caffeine immediately before initiating the short nap [46].

### Sport-performance’ level-based difference

There was no significant difference between the two sport-performance level sub-groups in sleep quality, insomnia severity, daytime sleepiness, negative emotions and QOL. However, international athletes reported a lower SE (with a small effect size) compared to world-class athletes. This may be explained by the higher reported number of soda beverages’ consumption per week, since higher soda consumption has been associated with higher ISI and PSQI scores (Figure 2). Indeed, sugar-sweetened beverage (i.e., including soda) consumption is negatively associated with TST in adolescents and adults, with a higher negative impact on adolescents [12].

### Discipline-based difference

Athletes from team- and individual- sports reported going to bed at the same time; however, individual sports athletes wake up earlier, leading to a shorter TST. This aligns with an earlier report, indicating that wake-up time is the most important predictor of TST in Australian elite athletes [2, 6, 33]. In fact,70% of individual-sports athletes reported 7 hours of TST or less, compared to only 40% of team-sports athletes (Table 2a). The proportion of severe daytime sleepiness was higher (18%) in individual-sport athletes compared to (2%) in teamsport athletes (Table 1). This may explain the higher daytime sleepiness and longer and more frequent daytime naps reported by individual-sports athletes. Although the difference in PSQI scores was not statistically significant, earlier reports showed a lower sleep quality in individuals compared to team-sport athletes [33].

### Satisfaction-based difference

This study is the first to investigate the association of career satisfaction with sleep quality in elite athletes. Those who were unsatisfied with their career at the time of our investigation showed shorter TST, higher insomnia severity and daytime sleepiness and lower QOL (Figure 1). In addition, 77% of the unsatisfied group reported sleeping 7 hours or less, compared to only 41% in the satisfied group. Moreover, unsatisfied athletes reported higher caffeine consumption probably to overcome the higher daytime sleepiness, which was associated with higher negative emotions and lower sleep quality (Figure 2a, 3a). A 2023 meta-analysis reported that caffeine consumption reduced TST in a dose and timing manner, reducing SOL independently of the dose and the time of caffeine consumption [13]. Interestingly, unsatisfied athletes, despite their higher daytime sleepiness compared to satisfied athletes, did not report higher desired sleep duration, nor longer daytime naps. Speculatively, the lower QOL subscales (psychological, social and environmental health) seen in the unsatisfied group may be resulting from the longer training duration (Table 2b), without an adequate amount of sleep for psychological and physical recoveries.

### Strengths and limitations

To the best of the authors’ knowledge, this study is the first to assess the relationship between sleep quality, negative emotions and the QOL in elite athletes, while taking into consideration several aspects of athletes’ health. We conducted this study in September 2022, implying that some athletes were in the post-season, while others were in the pre-season preparation phase. Clearly, there were differences in training volume among sub-groups (young vs. old; world class vs. international; and satisfied vs. unsatisfied athletes), but training volume was not associated with any of the dependent variables (i.e., sleep quality, negative emotions and QOL). As training intensity and/or volume may affect sleep quality [8, 14], it is unclear to what extent this factor may have influenced the current results. Moreover, all measurements of sleep, emotions, and QOL were based on athletes’ self-reports, which may over-/under-estimate certain results due to the athletes’ emotional state at the time of the survey. Consequently, the need for more objective approaches, such as physiological sleep measures (polysomnography, actigraphy, etc.), would provide more detail on sleep quality. The current study included athletes from only two team sports (volleyball and rugby), and most individual sports were combat sports (65%: 49 out of 76), implying the need to investigate the quality of sleep of elite athletes practising other sports’ disciplines. The results of this study must be interpreted with caution, taking into consideration the specificities of Tunisian athletes and their environment (e.g., the weather and the Mediterranean diet).

### Recommendations/Practical applications

Taking into consideration the interaction between sleep quality and the QOL, raising the importance of proper sleep quality and quantity in elite athletes seems to be of paramount importance [35]. The sensitisation effort should encompass not just athletes but also extend to training staff and organisational entities, because some sport-related (avoiding early/late training/competition, managing jet lag) aspects of sleep hygiene may be beyond athletes’ control. Nevertheless, athletes should be educated on non-sport related behaviours that impact sleep hygiene, including reducing caffeine and sugarsweetened beverage intake, minimising nighttime light exposure, and reserving the bed solely for sleep (and avoiding activities like eating, playing, or watching TV in bed) [1, 5, 15]. Sleep extension, either by extra nocturnal sleep [4] or by daytime napping [47, 48], might be the most important recommendation. In addition, maintaining regular, preferably early, bedtime enhances sleep consolidation and generates the optimal sleep propensity at the habitual sleep time [15].

## CONCLUSIONS

Our study, on a sample of Tunisian elite athletes, confirms the literature data indicating that the sleep quality of elite athletes is generally modest, with female, young, individual sports athletes, and athletes who are unsatisfied with their career, reporting an even lower sleep quality. These subgroups of athletes take naps more frequently with longer durations. Furthermore, bidirectional relationships were found between low sleep quality, low mental health, and low QOL in these elite athletes. Finally, a longer career duration is associated with a lower quality of life in elite athletes.

## Data Availability

All data are stored on institutional servers of the corresponding author and available for any reasonable request.
